# Targeted therapy for mTORC1-driven tumours through HDAC inhibition by exploiting innate vulnerability of mTORC1 hyper-activation

**DOI:** 10.1038/s41416-020-0839-1

**Published:** 2020-04-27

**Authors:** Fuchun Yang, Shaogang Sun, Chenran Wang, Michael Haas, Syn Yeo, Jun-Lin Guan

**Affiliations:** Department of Cancer Biology, University of Cincinnati College of Medicine, Cincinnati, OH 45267 USA

**Keywords:** Autophagy, Targeted therapies

## Abstract

**Backgound:**

The mechanistic target of rapamycin complex 1 (mTORC1) is important in the development and progression of many cancers. Targeted cancer therapy using mTORC1 inhibitors is used for treatment of cancers; however, their clinical efficacies are still limited.

**Methods:**

We recently created a new mouse model for human lymphangiosarcoma by deleting *Tsc1* in endothelial cells and consequent hyper-activation of mTORC1. Using *Tsc1*^iΔEC^ tumour cells from this mouse model, we assessed the efficacies of histone deacetylase (HDAC) inhibitors as anti-tumour agents for mTORC1-driven tumours.

**Results:**

Unlike the cytostatic effect of mTORC1 inhibitors, HDAC inhibitors induced *Tsc1*^iΔEC^ tumour cell death in vitro and their growth in vivo. Analysis of several HDAC inhibitors suggested stronger anti-tumour activity of class I HDAC inhibitor than class IIa or class IIb inhibitors, but these or pan HDAC inhibitor SAHA did not affect mTORC1 activation in these cells. Moreover, HDAC inhibitor-induced cell death required elevated autophagy, but was not affected by disrupting caspase-dependent apoptosis pathways. We also observed increased reactive oxygen species and endoplasmic reticulum stress in SAHA-treated tumour cells, suggesting their contribution to autophagic cell death, which were dependent on mTORC1 hyper-activation.

**Conclusion:**

These studies suggest a potential new treatment strategy for mTORC1-driven cancers like lymphangiosarcoma through an alternative mechanism.

## Background

Mechanistic target of rapamycin complex 1 (mTORC1) is a master regulator that controls cell growth, proliferation, autophagy and metabolism via its diverse downstream signalling pathways.^1,2^ Dysfunction of mTORC1 signalling has been implicated in many diseases, including human angiosarcoma and other cancers.^3–5^ mTORC1 is negatively regulated by tuberous sclerosis complex (TSC) tumour suppressor proteins TSC1 and TSC2.^6^ We recently created an inducible endothelial cell (EC)-specific *Tsc1* deletion mouse model (i.e. *Tsc1*^iΔEC^ mice) and found that hyper-activation of mTORC1 signalling in ECs resulted in the development of lymphatic malformation (LM) and progression to vascular tumours that recapitulate salient features of human lymphangiosarcoma (LAS), including local invasion and systemic metastasis.^5^ We also developed a vascular tumour cell line from *Tsc1*^iΔEC^ mice, which is capable of inducing vascular tumour formation upon xenograft transplantation in recipient mice. This new mouse model and vascular tumour cells offer unique opportunities to investigate the mechanisms of angiosarcomas to develop effective therapeutic strategies for the deadly disease.

Targeting mTOR signalling for therapy using rapamycin and its analogues for mTOR hyper-activated tumours has shown beneficial effects in preclinical models, including in our vascular tumour mouse model, and some clinical trials.^5,7,8^ However, mTOR-targeted cancer therapies still have significant challenges due to limited activities in clinical treatment.^9,10^ Histone deacetylases (HDACs) are enzymes that remove acetyl groups from acetyl-lysine residues in histones and various nonhistone proteins, which play important roles in numerous biological processes primarily through their regulation of chromatin structure and transcription.^11^ Because of essential roles of HDAC in many cancer cells, HDAC inhibitors (HDACi) have been used for preclinical and clinical trials in cancer therapies.^12–14^ Initially used for blood cancer treatment,^15,16^ HDACi also showed potential therapeutic efficacy in some solid cancers in recent studies.^17,18^ They have been shown to induce cancer cell death, cell cycle arrest, senescence and differentiation, as well as increase in cancer immunogenicity, via multiple mechanisms.^19^ One attractive feature of HDACi is a direct correlation between their ability to cause cell death and anti-cancer efficacy for tumour regression.^20^ Also, inhibitors for different HDAC class/members may exert their anti-cancer activities through different mechanisms.^21–24^ For example, a selective class IIa HDACi, TMP195, was recently shown to alter tumour microenvironment to enhance chemotherapy and immune checkpoint therapy for their anti-cancer efficacy in breast cancer models.^25^ Another advantage of HDACi in cancer therapy is their apparently preferential inhibitory activity towards many cancer cells relative to normal cells.^26,27^

In this study, we investigated the potential efficacy of HDAC inhibition for mTORC1-driven/hyper-activated tumours mainly using the newly developed *Tsc1*^iΔEC^ tumour cells. HDACi induced *Tsc1*^iΔEC^ tumour cell death in vitro and reduced their growth in vivo, without altering mTORC1 hyper-activation per se. Interestingly, HDAC inhibition-induced tumour cell death required elevated autophagy, and correlated with increased reactive oxygen species (ROS) and endoplasmic reticulum (ER) stress of the tumour cells with mTORC1 hyper-activation. We further demonstrated that mTORC1 inactivation prevented HDACi-induced cell death through reduced ROS and ER stress in *Tsc1*^iΔEC^ tumour cells. These studies suggest a potential new targeted therapy for mTORC1-driven or hyper-activated tumours through a mechanism different from directly blocking mTOR signalling.

## Methods

### Generation and culture of *Tsc1*^iΔEC^ vascular tumour cells

Vascular tumour cells were isolated from the cutaneous tumours in tamoxifen-induced Tsc1f/f; Scl-Cre mouse, and then transduced with hRasV12-GFP and E1A to establish immortalised *Tsc1*^iΔEC^ tumour cells, as described previously.^5,28^ LN229 cells (ATCC® CRL-2611^TM^) were obtained from Dr. David R. Plas (University of Cincinnati College of Medicine) as a gift. LEF2 cells were initially obtained as a gift from Dr. Kun-Liang Guan (University of California at San Diego) and as described previously.^29^ All cells were cultured in Dulbecco’s modified Eagle’s medium containing 10% foetal bovine serum supplied with 1% penicillin–streptomycin and grew in 5% CO_2_ at 37 °C. All cells were tested for negative mycoplasma contamination.

### Plasmids and generation of stable cell lines

Single guide RNA targeting mouse Fip200 5′-TTCTCTAGAAATAACACTAA-3′ was subcloned into pSpCas9(BB)-2A-GFP (PX458) (Addgene #48138) CRISPR-knockout (KO) vector according to the protocol previously reported.^30^ Rictor shRNA-5 (TRCN0000123395) and shRNA-6 (TRCN0000123396) were obtained from Sigma-Aldrich MISSION® shRNA (short hairpin RNA). Non-target shRNA (#1864), raptor shRNA-1 (#21339) and shRNA-2 (#21340) were purchased from Addgene.

*Fip200* KO in *Tsc1*^iΔEC^ cells was generated by transiently transfecting PX458-sgFip200 into *Tsc1*^iΔEC^ with Lipofectamine 3000 (Life Science Technology) according to the reagent protocol. Two days after transfection, single cells were seeded into 96-well plate by limited dilution method. Fip200 KO efficiencies in single clone expanded cells were confirmed by western blotting. pHAGE-CMV-Rheb (S16H)-IRES-eGFP-W (plasmid #32520) was purchased from Addgene. Lentiviral production containing Rheb (S16H) was transduced into LN229, and then EGFP (enhanced green fluorescent protein)-positive cells were sorted with flow cytometry and expanded.

All lentiviral productions were generated by transfecting into HEK293T cells with psPax2, pMD2.G and lentiviral plasmids, as described previously.^31^ *Tsc1*^iΔEC^ cells were then transduced by different lentivirus containing specific shRNA, followed by puromycin selection to generate stable knockdown tumour cells.

### In vitro and in vivo treatment of compounds

SAHA (catalogue no. S0147), CI994 (catalogue no. S2818) and Tubstatin A (catalogue no. S8049) were purchased from Selleckchem. TMP269 (catalogue no. 17738), rapamycin (catalogue no. 13346), Torin1 (catalogue no. 10997), LY294002 (catalogue no. 70920), MK2206 (catalogue no. 11593) and Z-VAD(OMe)-FMK (catalogue no. 14463) and *N*-acetyl-l-cysteine (catalogue no. 20261) were purchased from Cayman Chemical. Spautin1 (catalogue no. SML0440) was purchased from Sigma-Aldrich. For in vitro treatment, concentrations and time of the compounds are shown in the figure legends. For in vivo treatment, athymic nude mice (6-week-old female) were transplanted by subcutaneous injection of 1 × 10^6^ *Tsc1*^iΔEC^ tumour cells per flank. No anaesthesia or analgesia was administered upon transplantation, as approved by the Institutional Animal Care and Use Committee guidelines at the University of Cincinnati (Cincinnati, OH). When tumour volume grew into 100 mm^3^, mice were intraperitoneally injected with SAHA (50 mg/kg), CI994 (100 mg/kg) and Tubstatin A (100 mg/kg) daily for total 14 days, 10% dimethyl sulfoxide (DMSO) plus 45% PEG400 as vehicle. The formulation and dose of the compounds used were based on compound solubility according to the company protocol, maximum injection volume of single dose and data from our in vitro experiments. Treatments were carried out in conventional housing room throughout the experiment. Mice were fed with chow and water ad libitum. All mice were finally killed with medical carbon dioxide and cervical dislocation at the end point time. Tumour size was measured at the indicated time points (every 2 days) and calculated according to the formula: volume = (length × width^2^)/2. Tumour growth curves and tumour volumes were analysed using the GraphPad Prism 7 software. Mice were housed and handled according to local, state and federal regulations. All experimental procedures were carried out according to protocols approved by the Institutional Animal Care and Use Committee at the University of Cincinnati (Cincinnati, OH).

### Cell proliferation, viability, colony formation assay and cell death assay

A total of 2 × 10^4^ cells were seeded in 6-well plates with triplicates for each experimental group. Next day, the compound or vehicle was added into the culture medium. Cell count was measured as the indicated days with Countessmy of Life Science Technology. Cell viability was assessed by CellTiter-Glo Luminescent Cell Viability Assay Kit (Promega) and Cell Counting Kit-8 (CCK-8, Dojindo Molecular Technologies) according to their manual protocols. A total of 2 × 10^4^ cells were seeded in 96-well plate with triplicates for each experimental group; the next day, cells were treated by SAHA for 24 h. For colony formation assay, 500 cells were seeded in 6-well plates with triplicates, then treated with compounds the next day and finally stained with 2% crystal violet solution after 6 days of treatment. For cell death analysis, 2 × 10^5^cells were seeded into 12-well plates with triplicates and then treated with compounds for 18 h. Propidium iodide (PI) (BD 51-66211E) at 5 μM was incubated according to the protocol, and then cells were analysed by BD LSRFortessa flow cytometer system.

### ROS detection

For analysis of ROS of tumour cells in vitro, 2 × 10^5^ cells were seeded in 12-well plates with triplicates for each group and treated with compounds for 18 h. Live cells were collected and incubated with MitoSOX Red (Molecular Probes, M36008) at 5 μg/ml for 15 min at room temperature in dark, and then cells were analysed by BD LSRFortessa flow cytometer system.

### RT-qPCR

Total RNAs were isolated from cells with GeneJET RNA Purification Kit (Thermo Scientific #K0731) according to the user manual. Reverse transcription complementary DNAs (cDNAs) were synthesised with iScript cDNA Synthesis Kit (Bio-Rad #1708891). Real-time PCR was performed with iQ SYBR Green Supermix Kit (Bio-Rad #170-8880). Expression values were normalised to β-actin. The primers were obtained from PrimerBank (https://pga.mgh.harvard.edu/primerbank/) unless specific references were cited. The specificity of all primers was validated with their dissociation curves. The sequence (5′–3′) and amplicon length of each pair of primers are listed as follows:

*Atg5* (120 bp): TGTGCTTCGAGATGTGTGGTT (fwd); GTCAAATAGCTGACTCTTGGCAA (rev). *Atg16L1* (153 bp): CAGGCGTTCGAGGAGATCATT (fwd); ACTATCATTCCACGCACCATCA (rev). *Atg13* (167 bp): CCAGGCTCGACTTGGAGAAAA (fwd); AGATTTCCACACACATAGATCGC (rev). *Fip200* (143 bp): GACACTGAGCTAACTGTGCAA (fwd); GCGCTGTAAGTACACACTCTTC (rev). *Ulk1* (190 bp): AAGTTCGAGTTCTCTCGCAAG (fwd); CGATGTTTTCGTGCTTTAGTTCC (rev). *Sqstm1* (172 bp):^32^ AGCACAGGCACAGAAGACAA (fwd); AGCAGTTTCCCGACTCCATC (rev). *Becn1* (197 bp): ATGGAGGGGTCTAAGGCGTC (fwd); TCCTCTCCTGAGTTAGCCTCT (rev). *Atg7* (161 bp): GTTCGCCCCCTTTAATAGTGC (fwd); TGAACTCCAACGTCAAGCGG (rev). *Map1lc3a* (153 bp): GACCGCTGTAAGGAGGTGC (fwd); CTTGACCAACTCGCTCATGTTA (rev). *Bip* (93 bp):^33^ GCTTCGTGTCTCCTCCTGAC (fwd); TAGGAGTCCAGCAACAGGCT (rev). *Bim* (160 bp):^34^ GGAGATACGGATTGCACAGGAG (fwd); CCTTCTCCATACCAGACGGAAG (rev). *ERp72* (96  bp):^33^ TTCCACGTGATGGATGTTCAG (fwd); AGTCTTACGATGGCCCACCA (rev). *β-Actin* (154  bp): GGCTGTATTCCCCTCCATCG (fwd); CCAGTTGGTAACAATGCCATGT (rev).

### Chromatin immunoprecipitation

Chromatin immunoprecipitation (ChIP) assay was performed using EZ ChIP^TM^ Kit (Millipore, catalogue #17-371) according to the manufacturer’s protocol. A total of 1 × 10^7^ *Tsc1*^iΔEC^ tumour cells were seeded into 60 mm dish. The next day, vehicle or compounds were added into the culture medium at various concentrations for 24 h. Then, cells in each dish were crosslinked in 1% formaldehyde and lysed in SDS (sodium dodecyl sulfate) lysis buffer. Chromatin was sheared by Ultrasonic Processor on wet ice. Protein G Agarose and 3–4 μg acetyl-H3K9 Ab (Cell Signalling Tech) were used to precipitate DNA–protein overnight at 4 °C, using normal rabbit serum (CST) as a negative control. Reverse crosslinks were incubated overnight at 65 °C. DNA was purified using spin columns. Real-time PCR (Applied Biosystems, PowerUp™ SYBR™ Green Master Mix, A25742) was performed to analyse interactions between acetyl-H3K9 and select autophagy-related genes. The precipitated DNA samples were used as PCR templates, and fold-change values were normalised to the input of each group. The primers were obtained from previous reported references.^35,36^ The specificity of all the primers was validated with their dissociation curves in our experiments. The sequence and amplicon length of promoter-specific primers are listed as follows: *Ulk1* (182 bp): AGCCCCAACTGCCGGCTAGAA (fwd); TGTGATGTCTGACCCAGTGGCCC (rev). *Atg5* (200 bp): TGGCAGGTGAGAATGTTACAC (fwd); GGCTTTGGAGGATGTTGTCTGT (rev). *Atg7* (72 bp): GCAAAGCAAAGGTAAGCAAT (fwd); GCTGTTCTGGACCTGTATAC (rev). *Becn1* (81  bp): TAGACGGTCCACTCAAGATT (fwd); AACAAACAAACAACACCCTG (rev). *Map1lc3a* (70 bp): TTTCAGAGGACCTAAGCTTG (fwd); AACATAGCATCGGATTTCCT (rev).

### Western blotting

Lysates were prepared from tumour cells and analysed by western blotting. Primary antibodies include cleaved caspase-3 (1:2000, Cell Signalling #9661), phospho-rpS6 (Ser240/244) (1:5000, Cell Signalling #5364), rpS6 (1:5000, Cell Signalling #2217), phospho-p70 S6K (Thr389) (1:1000, Cell Signalling #9205), p70 S6K (1:1000, Santa Cruz #sc-230), C/EBP homologous protein (Chop) (1:1000 Cell Signalling #2895), FIP200 (1:1000, Cell Signalling #12436), p62 (1:2000, Cell Signalling #39749), LC3B (1:2000 Cell Signalling #3868), Raptor (1:1000, Cell Signalling #2280), Rictor (1:1000, Cell Signalling #2114), acetyl-histone H3(Lys9) (1:2000, Cell Signalling #9649), vinculin (1:10,000, Sigma-Aldrich #V4505) and NRF2 (1:1000, GeneTex #103322). Horse radish peroxidase (HRP)-linked anti-rabbit immunoglobulin G (IgG) or anti-Mouse IgG (1:10,000, Cell Signalling) were used as secondary antibodies. WesternBright ECL HRP substrate (Advansta) was used for signal imaging.

### Immunofluorescence

A total of 2 × 10^4^ cells were seeded into an 8-well chamber and treated with compounds the next day for 24 h. Then, cells were permeabilised with pure methanol for 10 min on ice, followed with 1% bovine serum albumin blocking buffer for 1 h at room temperature. Anti-LC3B was incubated with cells in the blocking buffer overnight at 4 °C. Next, Alexa fluorescence 594 goat anti-rabbit was used for 1 h at room temperature, followed by ProLong® Gold Antifade Mountant. Images were acquired with Zeiss LSM confocal 710. LC3 puncta was counted using Image J1.52i (Wayne Rasband; NIH; USA).

### Histology and immunostaining

Mouse tumour tissues were kept in 4%  paraformaldehyde at 4 °C. The specimens were then processed, embedded in paraffin and sectioned at 5 μm. Heat-induced antigen retrieval was performed in citrate buffer using a pressure cooker. Sections were incubated with primary antibodies overnight at 4 °C. Primary antibodies used were cleaved caspase-3 (1:200, Cell Signalling #9661), phospho-histone H3 (Ser10) (1:100, Cell Signalling #9701), Ki67 (1:500, Spring Bioscience M3060) and LC3B (1:1600, Cell Signalling #3868). Sections were then incubated with biotinylated goat anti-rabbit IgG (for 1 h at room temperature), followed by VECTASTAIN ABC peroxidase and DAB staining (Vector Laboratories), and then counterstained with haematoxylin. Images were acquired with Olympus BX41 microscope. Six random fields from three different tissues in each group were quantified using the ImageJ software.

### Statistical analysis

Statistical significance was determined by unpaired two-tailed Student’s *t* test. *P* value < 0.05 was considered as statistical significance.

## Results

### HDACi induce *Tsc1*^iΔEC^ tumour cell death in vitro and inhibit tumour growth in vivo

**Fig. 1:**
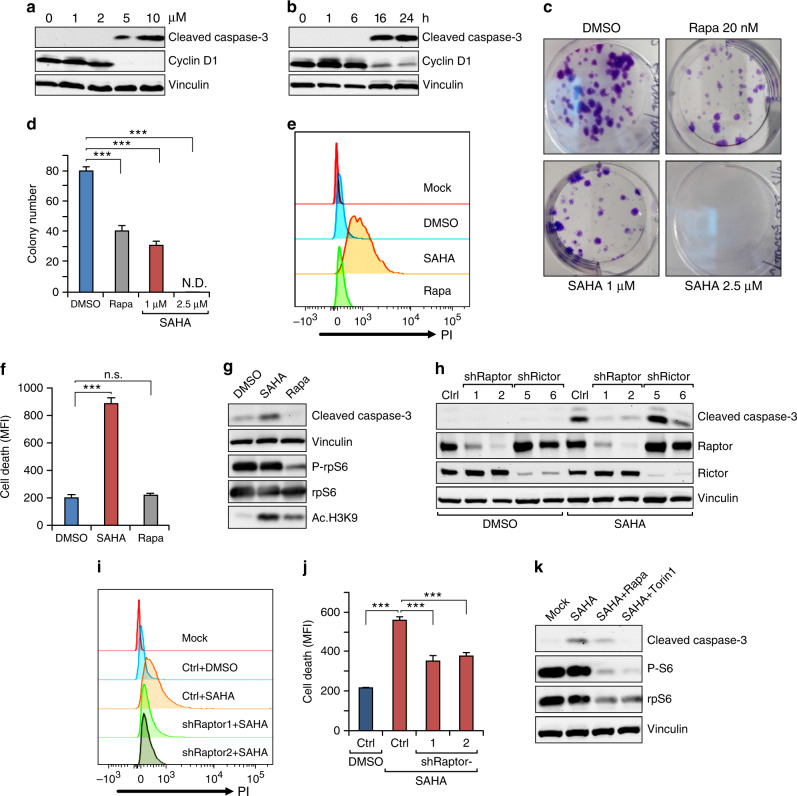
HDAC inhibitors treatment induces *Tsc1*^iΔEC^ tumour cells death. **a**, **b** Western blot analysis of cleaved caspase-3 and cyclin D1 in *Tsc1*^iΔEC^ tumour cells treated with SAHA at the indicated concentrations for 24 h (**a**) or at 5 μM for the indicated times (**b**). **c**, **d** Colony formation assay of *Tsc1*^iΔEC^ tumour cells treated as indicated for 7 days. Representative images (**c**) and mean ± SD of the colony numbers (**d**) are shown. **e**, **f** Cell death analysis of *Tsc1*^iΔEC^ tumour cells treated with SAHA (5 μM), rapamycin (100 nM) or DMSO for 18 h. Cells were stained with PI and analysed by flow cytometry, with representative results (**e**) and mean ± SD of MFI (**f**) shown. **g** Western blot analysis of cleaved caspase-3 and phospho-rpS6, as well as acetyl-histone H3 at lysine 9 (Ac-H3K9) upon SAHA (5 μM), rapamycin (100 nM), or DMSO treatment for 24 h. **h** Western blot analysis of Cl. Casp3 in *Tsc1*^iΔEC^ tumour cells with raptor or rictor shRNA knockdown and non-targeting shRNA (shNT) as control (Ctrl). **i**, **j** Cell death analysis of *Tsc1*^iΔEC^ Ctrl (shNT) and raptor knockdown (shRaptor) tumour cells upon SAHA (5 μM) or DMSO treatment. Cells were stained with PI and analysed by flow cytometry, with representative results (**i**) and mean ± SD of MFI (**j**) shown. **k** Western blot analysis of Cl. Casp3 and P-rpS6 upon DMSO, SAHA treatment or combination treatment with rapamycin or Torin1. In all western blots, vinculin is used as an endogenous loading control. For all figures, *n* = 3. ***P < 0.001; n.s., no significance; N.D., nondetectable. MFI mean fluorescence intensity.

To determine potential anti-cancer efficacy of HDACi on vascular tumour cells, we first treated *Tsc1*^iΔEC^ tumour cells with SAHA (a pan-HDACi) and examined effects on cell apoptosis and colony formation. While no effect at 2 μM or lower dose, treatment for 24 h with SAHA at 5 and 10 μM^37^ induced significant cell apoptosis, as measured by cleaved caspase-3 (Fig. 1a). Time-course analysis showed induction of apoptosis at 16 h after treatment with 5 μM SAHA (Fig. 1b). We also observed corresponding decreased levels of cyclin D1 (Fig. 1a, b), consistent with reduced cell proliferation and apoptosis. Moreover, inhibition of *Tsc1*^iΔEC^ tumour cell proliferation was also observed by several direct cellular assays, including colony formation (Fig. 1c, d), cell count and viability measurements (Supplementary Fig. S1). Interestingly, SAHA treatment, but not mTORC1 inhibitor rapamycin, induced significant tumour cell death under our experimental conditions (Fig. 1e–g). Further, SAHA did not affect mTORC1 activation in the tumour cells, whereas rapamycin inhibited it as expected (Fig. 1g). Inhibition of HDACs by SAHA led to increased histone H3K9 acetylation, as expected, while rapamycin also increased it moderately. These findings suggest that HDACi may play an anti-tumour role by inducing *Tsc1*^iΔEC^ tumour cell death and cell cycle arrest through mechanisms independent of mTORC1 inhibition and possibly to a greater extent than mTORC1 inhibition by rapamycin.

To explore potential specificity of HDACi-induced cell death in tumour cells with hyper-activated mTORC1, we examined the effect of SAHA on *Tsc1*^iΔEC^ tumour cells following shRNA knockdown of Raptor or Rictor to inhibit mTORC1 or mTORC2, respectively. We found that Raptor knockdown significantly decreased SAHA-induced cell death, as measured by levels of cleaved caspase-3, whereas Rictor knockdown did not have any effect (Fig. 1h). Neither induced cell death on their own in cells treated with DMSO. Measurement of cell death by PI staining, followed by flow cytometry analysis, also showed that Raptor knockdown decreased SAHA-induced cell death in *Tsc1*^iΔEC^ tumour cells (Fig. 1i, j). Consistent with these data, mTORC1 inhibition by rapamycin or Torin1 also reduced SAHA-induced cell death, as measured by cleaved caspase-3 levels (Fig. 1k). These results suggest that anti-tumour efficacy of HDACi is dependent on mTORC1 hyper-activation in *Tsc1*^iΔEC^ tumour cells.

**Fig. 2:**
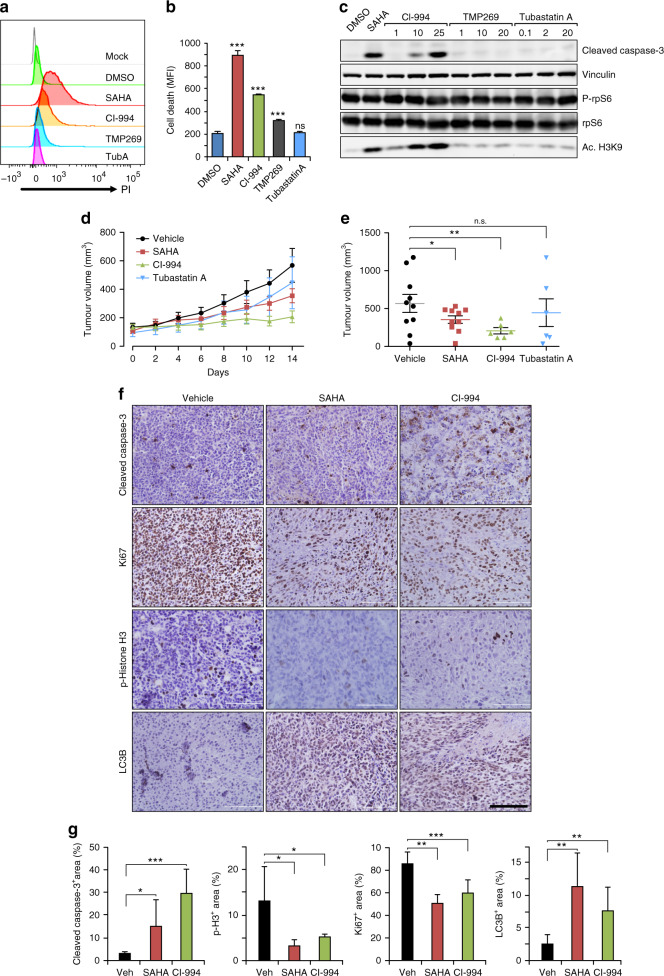
HDAC inhibitors have anti-tumour effects on *Tsc1*^iΔEC^ tumour in vivo. **a**, **b** Cell death analysis of *Tsc1*^iΔEC^ tumour cells upon SAHA (5 μM), CI994 (25 μM), TMP269 (20 μM), Tubastatin A (Tub A, 20 μM) or DMSO treatment for 18 h. Representative results (**a**) and mean ± SD of MFI (**b**) were shown. **c** Western blot analysis of Cl. Casp3, p-rpS6, rpS6 and Ace-H3K9 in *Tsc1*^iΔEC^ tumour cells upon SAHA (5 μM), CI994, TMP269, Tub A or DMSO treatment at the indicated concentrations for 24 h. **d** *Tsc1*^iΔEC^ tumour growth upon SAHA (50 mg/kg), CI994 (100 mg/kg), Tub A (100 mg/kg) or vehicle treatment for 14 days. Tumour volumes are shown as mean ± SEM; *n* = 3–5 mice (6–10 tumours). **e** *Tsc1*^iΔEC^ tumour volumes at day 14. *P < 0.05, **p < 0.01; n.s., no significance. **f**, **g** Immunohistochemistry (IHC) analysis of Cl. Casp3, phospho-histone H3 at Ser10 (p-histone H3), Ki67 and LC3B (**f**). Scale bar, 100 μm, ×40 magnification. Graphs depict the percentage of positive staining areas in each group (**g**) shown as mean ± SD. *P < 0.05, **p < 0.01, and ***p < 0.001.

We next tested the effect of several selective HDACi targeting different members of HDAC subfamily in comparison to the pan-HDACi SAHA. We found that class I HDACi CI994 (for HDAC1, 2, 3 and 8) induced significant amount, although less than SAHA, of cell death as measured by both PI staining and cleaved caspase-3 levels (Fig. 2a–c). Class IIa HDACi TMP269 (for HADC4, 5, 7 and 9) caused a small increase of cell death as measured by PI staining, but not based on the presence of cleaved caspase-3. Conversely, no apparent difference in PI staining or cleaved caspase-3 was observed in cells treated with the selective class IIb inhibitor Tubastatin A (for HDAC6). None of the inhibitors affected mTORC1 activation (Fig. 2c). While CI994 increased acetylation of H3K9 levels in a dose-dependent manner as expected, either TMP269 or Tubastatin A treatment led to consistent changes in H3K9 acetylation levels (Fig. 2c). These results suggested that SAHA treatment induced *Tsc1*^iΔEC^ tumour cell death primarily through class I HDACi targets.

To examine the effect of SAHA and CI994 in vivo, *Tsc1*^iΔEC^ tumour cells were transplanted into nude mice, and when tumours reached ~100 mm^3^ in volume (day 0), they were randomised into four groups treated daily with various inhibitors or vehicle. Consistent with data in vitro, treatment with either SAHA or CI994 significantly inhibited tumour growth, but Tubastatin A treatment did not (Fig. 2d, e). Interestingly, CI994 had a greater inhibitory effect than SAHA, although it was less effective to induce cell death in vitro. Analysis of tumour sections by haematoxylin and eosin staining verified significantly reduced tumour growth by SAHA or CI994 treatments (Supplementary Fig. S2). Immunohistochemistry analysis showed increased apoptosis, and decreased cell proliferation in tumours from recipient mice treated with SAHA or CI994 relative to vehicle-treated mice (Fig. 2f, g). Together, these results suggest that HDACi SAHA and CI994 have significant anti-tumour activities for vascular tumours that are potentially more effective than rapamycin by inducing cell death as well as inhibiting cell proliferation.

### SAHA-induced *Tsc1*^ΔEC^ tumour cell death requires FIP200-mediated autophagy

**Fig. 3:**
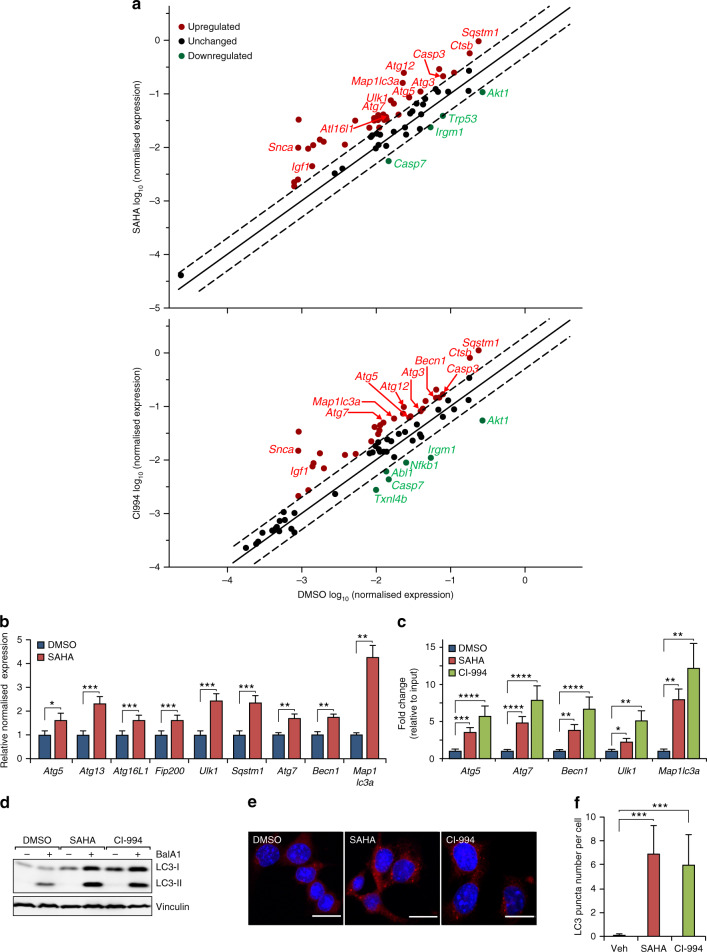
Autophagy activities are up-regulated by HDAC inhibitor treatment. **a** RT-qPCR gene expression analysis of cell death pathways with RT^2^ Profiler PCR Array Kit (Qiagen) upon SAHA (5 μM), CI994 (25 μM) or DMSO treatment of *Tsc1*^iΔEC^ tumour cells, with data shown as scatter plot. Fold regulation and significance thresholds were set at 2 and *p* < 0.05 respectively. **b** RT-qPCR gene expression analysis of autophagy-related genes shown as mean ± SEM. *n* = 3, *p < 0.05, **p < 0.01, and ***p < 0.001. **c** ChIP-qPCR analysis of acetyl-H3K9 binding to the promoters of autophagy-related genes shown as mean ± SEM. *n* = 3, *p < 0.05, **p < 0.01, ***p < 0.001, and ****p < 0.0001. **d** Western blot analysis of LC3-I to LC3-II conversion upon SAHA (5 μM), CI994 (25 μM) or DMSO treatment for 24 h with or without bafilomycin A1 (Bal A1, 200 nM) for the final 4 h, with vinculin as an endogenous loading control. **e**, **f** Immunofluorescence analysis of LC3 puncta in *Tsc1*^iΔEC^ upon SAHA (5 μM), CI994 (25 μM) or DMSO treatment for 24 h. Representative images are shown (**e**). Scale bar, 20 μm. Graph shows LC3 puncta number per cell (**f**) as mean ± SD. ***P < 0.001.

To explore the potential mechanisms of HDACi-induced *Tsc1*^iΔEC^ tumour cell death, we performed gene expression analyses after SAHA or CI994 treatment of these cells using quantitative reverse transcription PCR (RT-qPCR)-based cell death pathway array assay. By setting 2× difference as a threshold, we found 32 up-regulated genes and 4 down-regulated genes in *Tsc1*^iΔEC^ tumour cells after treatment with SAHA compared to vehicle-treated cells (Fig. 3a). Of the up-regulated genes, eight were apoptosis genes and nine were necrosis genes, which may contribute to the increased cell death observed after SAHA treatment (see Figs. 1, 2). Interestingly, the remaining 15 up-regulated genes were related to autophagy, which has been associated with causing cell death (i.e. autophagic cell death)^38^ as well as tumour cell survival and increased drug resistance.^39–41^ Also, CI994 treatment in *Tsc1*^iΔEC^ tumour cells caused the upregulation of 32 genes and the downregulation of 6 genes (Fig. 3a). Among the up-regulated genes, there were 9 apoptosis-related genes, 9 necrosis-related genes and 14 autophagy-related genes, including 3 that were both classified as autophagy and apoptosis-related genes. We therefore focused on the potential role of autophagy in inducing cell death. RT-qPCR verification of the nine autophagy-related genes using different specific primers for the same set of messenger RNAs confirmed increased expression of all these genes, including *Atg5*, *Atg13*, *Atg16L1*, *Fip200*, *Ulk1*, *Sqstm1*, *Atg7*, *Becn1* and *Map1lc3a* (Fig. 3b). Given the increased H3K9 acetylation upon treatments with SAHA or CI994 (see Figs. 1g, 2c), we examined H3K9AC mark on promoters of multiple autophagy-related genes by ChIP with anti-H3K9AC, followed by qPCR. We found that both SAHA and CI994 increased the levels of H3K9AC in promoters of *Atg5*, *Atg7*, *Becn1*, *Ulk1* and *Map1lc3a* genes (Fig. 3c), suggesting that HDAC inhibition may lead to the upregulation of these autophagy-related genes through epigenetic regulation for increased transcription. Consistent with the increased expression of multiple autophagy-related genes, we detected increased flux of LC3-I into LC3-II and more LC3 puncta in tumour cells treated with SAHA or CI994 compared to vehicle-treated cells (Fig. 3d–f). We also found increased levels of LC3B in tumour sections from recipient mice transplanted with *Tsc1*^iΔEC^ tumour cells that had been treated with SAHA or CI994 (see Fig. 2g), which is consistent with an increased autophagy in these cells after HDACi treatment.

**Fig. 4:**
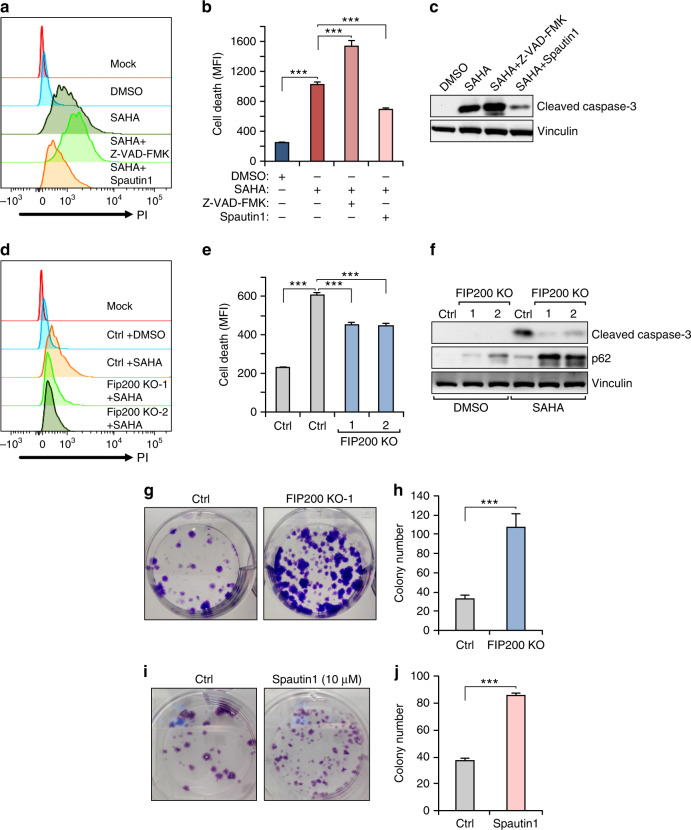
Autophagy is responsible for SAHA-induced *Tsc1*^iΔEC^ tumour cell death. **a**, **b** Cell death analysis of *Tsc1*^iΔEC^ tumour cells upon SAHA (5 μM) or DMSO treatment, or combination treatment with Z-VAD-FMK (10 μM) or spautin1 (10 μM). Cells were stained with PI and analysed by flow cytometry, with representative results (**a**) and mean ± SD of MFI (**b**) shown. **c** Western blot analysis of Cl. Casp3 in *Tsc1*^iΔEC^ tumour cells upon SAHA or DMSO treatment, or combination treatment with Z-VAD-FMK or spautin1, with vinculin as an endogenous loading control. **d**, **e** Cell death analysis of control and *Fip200* KO *Tsc1*^iΔEC^ tumour cells upon SAHA or DMSO treatment. Representative results (**d**) and mean ± SD of MFI (**e**) shown. **f** Western blotting analysis of Cl. Casp3 and p62 upon SAHA or DMSO treatment in control and *Fip200* KO *Tsc1*^iΔEC^. **g**, **h** Colony formation assay of control and *Fip200* KO *Tsc1*^iΔEC^ tumour cells treated with SAHA. Representative images (**g**) and mean ± SD of the colony numbers (**h**) are shown. **i**, **j** Colony formation assay of *Tsc1*^iΔEC^ tumour cells treated with SAHA (Ctrl) or SAHA and spautin1. Representative images (**i**) and mean ± SD of the colony numbers (**j**) shown. For all figures, *n* = 3. ***P < 0.001. MFI mean fluorescence intensity.

To investigate potential roles of the increased autophagy after HDACi treatment, we first examined the effect of blocking apoptosis by a pan-caspase inhibitor Z-VAD-FMK or autophagy inhibitor spautin1 on *Tsc1*^iΔEC^ tumour cells treated with SAHA. While Z-VAD-FMK did not prevent SAHA-induced cell death, autophagy inhibition by spautin1 partially decreased it as measured by several different assays (Fig. 4a–c), suggesting that *Tsc1*^iΔEC^ tumour cell death following SAHA treatment is primarily due to autophagic cell death rather than increased apoptosis. To further evaluate a role for autophagic cell death in SAHA-induced *Tsc1*^iΔEC^ tumour cell death, we used CRISPR-Cas9 to delete an essential autophagy gene *Fip200* in *Tsc1*^iΔEC^ tumour cells (Supplementary Fig. S3). Similar to spautin1, FIP200 deletion partially reduced SAHA-induced cell death (Fig. 4d–f). Moreover, both FIP200 KO and spautin1 increased colony formation of the SAHA-treated *Tsc1*^iΔEC^ tumour cells (Fig. 4g–j), likely as a result of reduced cell death. Together, these results suggested that HDAC inhibition-induced vascular tumour cell death is at least partially mediated by increased autophagic cell death.

### Excessive ROS and increased ER stress contribute to SAHA-induced autophagic cell death of *Tsc1*^iΔEC^ tumour cells

**Fig. 5:**
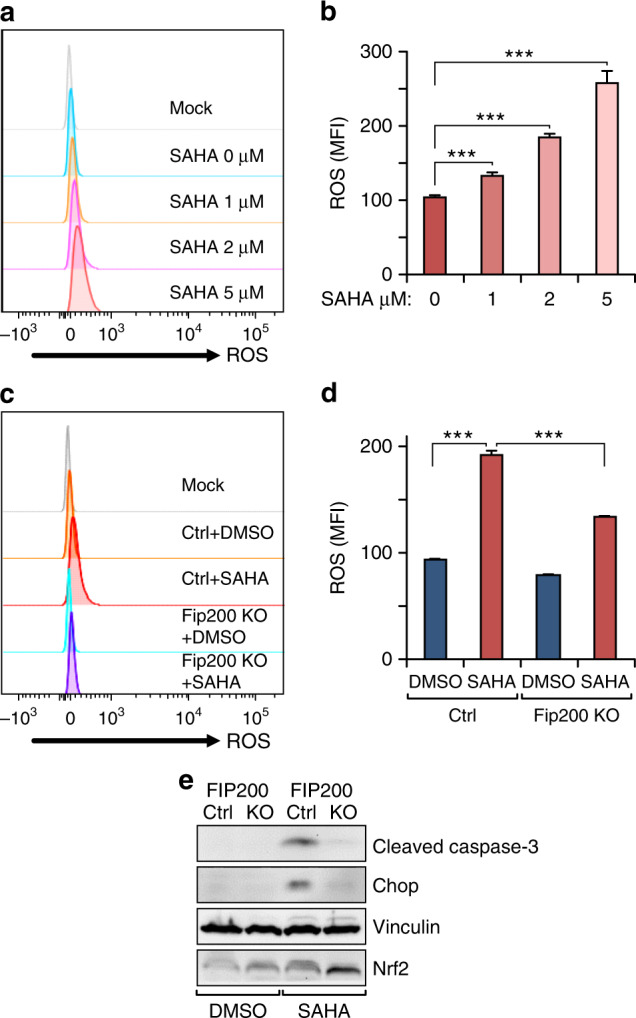
Autophagy mediates ROS and ER stress by SAHA treatment in *Tsc1*^iΔEC^ tumour cell death. **a**, **b** Flow cytometry analysis of ROS in *Tsc1*^iΔEC^ tumour cells with increasing SAHA concentrations. Cells were stained with MitoSOX Red and analysed by flow cytometry, with representative results (**a**) and mean ± SD of MFI (**b**) shown. **c**, **d** Flow cytometry analysis of ROS in control and *Fip200* KO *Tsc1*^iΔEC^ tumour cells upon SAHA (5 μM) or DMSO treatment. Cells were stained with MitoSOX Red and analysed by flow cytometry, with representative results (**c**) and mean ± SD of MFI (**d**) shown. **e** Western blotting analysis of Cl. Casp3, Chop, Ace-H3K9 and Nrf2 in control and *Fip200* KO *Tsc1*^iΔEC^ tumour cells upon SAHA or DMSO treatment, with vinculin as an endogenous loading control. For all figures, *n* = 3. ***P < 0.001. MFI mean fluorescence intensity.

Previous studies showed that SAHA treatment induced caspase-independent cell death of several different tumour cells accompanied with increased ROS production.^26,42,43^ We therefore examined ROS levels in SAHA-treated *Tsc1*^iΔEC^ tumour cells to gain further insights into mechanisms of autophagic cell death. We found that SAHA treatment increased ROS levels in *Tsc1*^iΔEC^ tumour cells in a dose-dependent manner (Fig. 5a, b). Further, SAHA-induced ROS increase was largely abolished in FIP200 KO-1 cells (Fig. 5c, d). We also measured activation of ER stress in SAHA-treated *Tsc1*^iΔEC^ tumour cells, as increased ROS has been reported to trigger downstream pathways leading to ER stress pathway activation.^44^ RT-qPCR showed increased levels of Bip and Bim, but not ERp72, which are associated with ER stress signalling (Supplementary Fig. S4). Furthermore, another important ER stress signalling downstream target, transcription factor Chop,^45^ was elevated in *Tsc1*^iΔEC^ tumour cells treated with SAHA, and this increase was abolished in FIP200 KO-1 cells (Fig. 5e). Previous studies showed that autophagy deficiency elicited cellular antioxidative activity through p62-Keap1-Nrf2 signalling.^46,47^ Our data showed that *Fip200* KO caused accumulation of p62 protein, especially by SAHA treatment (see Fig. 4f), so it is possible that increased p62 induces Nrf2 expression. Interestingly, we observed that Nrf2 expression elevated in *Fip200* KO *Tsc1*^iΔEC^ by SAHA treatment (Fig. 5e). These results suggest that increased ROS and ER stress may contribute to SAHA-induced autophagic cell death of vascular tumour cells.

**Fig. 6:**
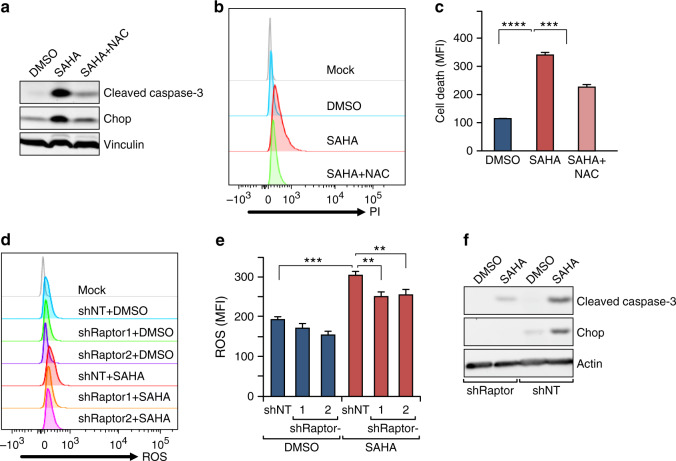
HDAC inhibitor selectively targets mTORC1 hyper-activated tumour cells. **a** Western blot analysis of cleaved caspase-3 and Chop expressions upon SAHA (5 μM), SAHA + NAC (5 mM) or DMSO treatment in *Tsc1*^*iΔEC*^ tumour cells. **b**, **c** Cell death analysis of *Tsc1*^iΔEC^ tumour cells upon SAHA, SAHA + NAC or DMSO treatment. Representative results (**b**) and mean ± SD of MFI (**c**) are shown. **d**, **e** Flow cytometry analysis of ROS in *Tsc1*^iΔEC^ Ctrl (shNT) and raptor knockdown (shRaptor) tumour cells upon SAHA or DMSO treatment. Cells were stained with MitoSOX Red and analysed by flow cytometry, with representative results (**d**) and mean ± SD of MFI (**e**) shown. **f** Western blot analysis of Chop expression in *Tsc1*^iΔEC^ Ctrl (shNT) and raptor knockdown (shRaptor) tumour cells. In all western blots, vinculin is used as an endogenous loading control. For all figures, *n* = 3. **P < 0.01, ***p < 0.001 and ****p < 0.0001.

To directly test whether the increased ROS is crucial for driving SAHA-induced cell death or just correlates with the death as a by-product of SAHA treatment, we employed a widely used anti-oxidant, *N*-acetyl-cysteine (NAC), to scavenge ROS and examine the effects on these cells. Indeed, treatment with NAC significantly reversed SAHA-induced cell death (Fig. 6a–c), suggesting that the generation of ROS was responsible at least partially in driving cell death by SAHA. Interestingly, the elevated level of Chop was reduced after NAC treatment (Fig. 6a), consistent with the possibility that ROS-triggered ER stress contributed to SAHA-induced death of vascular tumour cells. In addition, we found that Raptor knockdown in *Tsc1*^iΔEC^ tumour cells that reduced SAHA-induced cell death (see Fig. 1h–j) also decreased the ROS elevation (Fig. 6d, e) and abolished the induction of Chop expression (Fig. 6f) in these cells after SAHA treatment. These results provided further support that anti-tumour efficacy of HDACi is dependent on mTORC1 hyper-activation in *Tsc1*^iΔEC^ tumour cells, which were likely due to the preferential response of the increased ROS levels and ER stress in these cells.

### HDACi specifically target tumour cells with mTORC1 hyper-activation

To further explore the vulnerability of mTORC1 hyper-activated tumours to HDACi in other cancer cells, a human glioblastoma cell line LN229 was stably transfected with constitutively active Rheb (S16H), an upstream activator of mTOR, which activated mTOR signalling in neurons.^48^ As expected, the overexpression of Rheb (S16H) increased mTORC1 signalling, as indicated by increased phosphorylation of both p70 S6K and rpS6 (Supplementary Fig. S5A). Interestingly, LN229 tumour cells with active Rheb showed increased sensitivity to cell death induced by SAHA or CL994 as measured by cleaved capase-3 levels. We next assessed the effects of HDACi on rat renal carcinoma cells named LEF2, which has a Tsc2 loss-of-function mutation, with or without re-expression of Tsc2 to suppress mTORC1 activation. We found that while SAHA and CI994 both induced cell death of LEF2 control cells, they did not induce cell death for LEF2 cells with re-expression of Tsc2 (Supplementary Fig. S5B). Collectively, these data provide further support that HDACi could target mTORC1 hyper-activated tumours and had anti-tumour efficacies in mTORC1-driven/hyper-activated tumours without affecting mTORC1 activation per se.

Because Akt transcription in *Tsc1*^iΔEC^ was down-regulated by HDACi (see Fig. 3a), we also assessed the effects of combination treatments with SAHA and PI3K-Akt inhibitors (LY294002 or MK2206, respectively). As shown in Supplementary Fig. S6, we found that both combination treatments induced more cell death than single treatment by SAHA alone, and MK2206 showed stronger synergistic effect with SAHA for inducing tumour cell death. These data suggest that the combination of HDAC and Akt inhibitors treatment could have greater efficiency for the therapeutics of mTORC1-driven tumour cells.

## Discussion

LAS is angiosarcoma with lymphatic differentiation, which is a malignant type of vascular tumour originating from the transformation of ECs. It is a highly aggressive tumour without effective treatment currently, and the prognosis for patients is very poor, with a reported 5-year survival rate of ~10%.^49^ We have recently established the first mouse model that recapitulates all salient features of the human disease and showed critical importance of mTORC1 hyper-activation in driving its development and progression.^5^ Although rapamycin was effective in inhibiting tumour growth in this mouse model,^5^ translation of treatment with rapamycin or other mTOR inhibitors into human patients may be problematic due to their generally limited efficacies in many cancers for several reasons, including drug resistance and relapse.^10,50^ There are also concerns for rapamycin and its analogues on incomplete inhibition of mTOR and unequal effects on different mTOR substrates, as well as their acting generally through inhibition of proliferation rather than cytotoxicity to induce tumour cell death.^51,52^ In addition to the development of new generations of mTOR inhibitors and RapaLink-1 (a TORKi linked to rapamycin),^53,54^ combinatory targeting for mTORC1 and other pathways have been explored with success. It was shown recently that in mTORC1-driven tumours, combination of clinically approved rapamycin and mizoribine to inhibit mTORC1 and nucleotide synthesis, respectively, caused DNA replication stress and induced tumour cell apoptosis.^55^ In this study, we show that HDACi single treatment induced *Tsc1*^iΔEC^ tumour cell death in vitro and tumour growth in vivo. HDACi-induced tumour cell death is dependent on the hyper-activation of mTORC1 (i.e. inhibiting mTORC1 reduced HDACi efficacy, rather than synergise with it), thus revealing a new and independent mechanism of blocking mTORC1-driven tumours. This therapeutic strategy that exploit vulnerability of oxidative stress by mTORC1 hyper-activation for HDACi to induce tumour cell death could potentially be applied for other cancers too.

Autophagy has been shown to promote tumour cell survival or cell death in various context through different mechanisms.^39,56^ Whereas a number of studies including ours suggested pro-tumorigenic roles of autophagy in breast and other cancers,^28,57,58^ our findings suggested that increased autophagy upon HDACi treatment promoted *Tsc1*^iΔEC^ tumour cell death rather than enhancing drug resistance to help tumour cell survival as we observed previously in a breast cancer model after paclitaxel treatment.^59^ These results are consistent with previous studies showing that HDACi treatment can cause apoptosis and autophagic cell death in cancer cells.^60,61^ Other recent studies suggested that autophagy inhibition by Atg5 knockdown or chloroquine can enhance HDACi-induced cell death in glioblastoma cells or acute myeloid leukaemia cells.^38,62^ Similar to our observations, SAHA treatment was found previously to increase autophagy in glioblastoma cells and mouse embryonic fibroblasts (MEFs).^38^ In contrast to these studies, another report showed that the HDACi MGC0103 induced B-cell chronic lymphocytic leukaemia cell death by inactivating autophagy and activating PI3K/AKT/mTOR signalling pathway.^63^ Further studies will be required to fully understand the differential role of autophagy in HDACi-induced tumour cell death in these different contexts.

The mechanisms by which HDACi increased autophagy to trigger *Tsc1*^iΔEC^ tumour cell death are not fully understood at present. It was reported previously that SAHA treatment increased autophagy by inhibiting mTORC1 in MEFs.^38^ However, we found that SAHA increased autophagy without affecting mTORC1 activity in *Tsc1*^iΔEC^ tumour cells (see Figs. 1g, k, 2c). On the other hand, our observation of elevated ROS and ER stress that were correlated with the increased autophagy upon HDACi treatment and the preferential changes in the tumour cells with mTORC1 hyper-activation suggest a role for the increased ROS and ER stress contributing to autophagy induction and tumour cell death, which are consistent with several previous reports.^64,65^

Another interesting and not fully explained observation in this study is the paradoxical roles of mTORC1 hyper-activation in *Tsc1*^iΔEC^ tumour cells and their responses to HDACi. On the one hand, *Tsc1*^iΔEC^ tumours are driven by mTORC1 hyper-activation and can be inhibited by rapamycin^5^ (Fig. 1g, this study). On the other hand, mTORC1 inhibition did not synergise with HDACi to increase tumour cell death, but rather could alleviate HDACi-induced cell death. These data suggest that HDACi and mTORC1 inhibitors target different pathways, which are not only non-synergistic but also possibly antagonistic to each other, as our studies suggested that mTORC1 activation confers a synthetic lethal dependence on HDAC activity. Indeed, our previous studies showed that tumour cells from *Tsc1*^iΔEC^ mice, but not Tsc1-null ECs of non-tumour tissue (i.e. lung) of the same mice, readily form tumours when transplanted in nude mice.^5^ These results suggest that, while it is required, *Tsc1* deletion and consequent mTORC1 hyper-activation alone may not be sufficient to induce and maintain LAS. Thus, mTORC1 hyper-activation may require secondary mutations in other genes to induce and/or maintain LAS. Interestingly, in some clinical cases, malignant transformation of vascular malformation and benign tumours to angiosarcoma was accompanied with secondary mutations,^66,67^ although whether such secondary mutations contribute to the development and maintenance of angiosarcoma is not clear. It will be highly interesting to determine whether HDACi may target pathways driven by such secondary mutations and mechanisms involved in future studies.

## Supplementary information

Supplementary Information

## Data Availability

All original data and materials generated during the current study are available from the corresponding author upon reasonable request.
