# Supplementary material for: Targeted therapy for mTORC1-driven tumours through HDAC inhibition by exploiting innate vulnerability of mTORC1 hyper-activation
Source: Br J Cancer. 2020 Apr 27;122(12):1791–802. doi: 10.1038/s41416-020-0839-1 (PMC7283252; doi:10.1038/s41416-020-0839-1)
Supplement: Supplementary file 1 — Supplementary Information [file 41416_2020_839_MOESM1_ESM.pdf]

Legends for Supplemental figures

Figure S1.

A, *Tsc1*$^{i\Delta EC}$ tumor cell growth upon SAHA (2.5 μM) or DMSO treatment for 7 days (n = 3; ***, p < 0.001). **B,** *Tsc1*$^{i\Delta EC}$ tumor cell viability was assessed by luminescence upon SAHA (5 μM) or DMSO treatment for 3 days. *n* = 3. ***p < 0.001. **C,** *Tsc1*$^{i\Delta EC}$ tumor cell viability was measured at 450 nm absorbance upon SAHA (5μM) or DMSO treatment for 2 days. *n* = 3. ****p<0.0001.

Figure S2.

Image of representative xenograft tumor sections from mice treated with vehicle, SAHA or CI994. Scale bars are 5 mm.

Figure S3.

Western blot analysis of FIP200 expression in Ctrl and Fip200 KO *Tsc1*$^{i\Delta EC}$ tumor cells.

Figure S4.

RT-qPCR gene expression analysis of ER stress-related genes upon SAHA (5 μM) or DMSO treatment. Data are shown as mean ± SD. *n* = 3. ***p < 0.001; n.s., no significance.

Figure S5.

A, Western blotting analysis of Cl. Casp3, P-p70 S6K and P-rpS6 in LN229 tumor cells upon DMSO, SAHA (5μM) or CI-994 (25μM) treatment. **B,** Western blotting analysis of Cl. Casp3, P-p70 S6K and P-rpS6 in LEF tumor cells upon DMSO, SAHA (5μM) or CI994 (25μM) treatment. In all experiments, vinculin as an endogenous loading control.

Figure S6.

A and **B,** Cell death analysis of *Tsc1*$^{i\Delta EC}$ tumor cells upon SAHA (5 μM), SAHA (5 μM) + LY294002 (40 μM), SAHA (5 μM) + MK2206 (10 μM) or DMSO treatment for 18h. Cells were stained with PI and analyzed by flow cytometry. Representative results (**A**) and mean ± SD of MFI (**B**) shown. n=3. $^{**}p < 0.01$; $^{***}p < 0.001$.

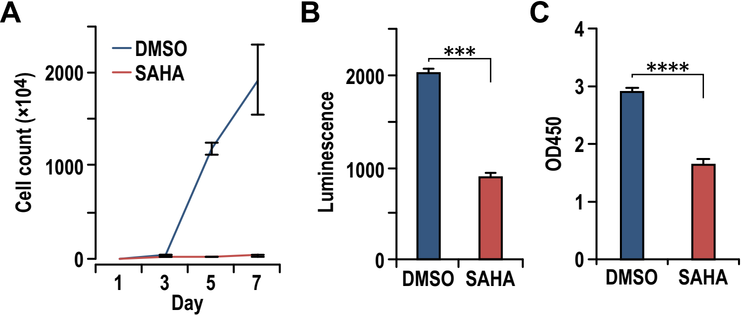
A
DMSO
SAHA
Cell count (×10^4)
0
1000
2000
1
3
5
7
Day
B
Luminescence
***
0
1000
2000
DMSO
SAHA
C
OD450
****
0
1
2
3
4
DMSO
SAHA

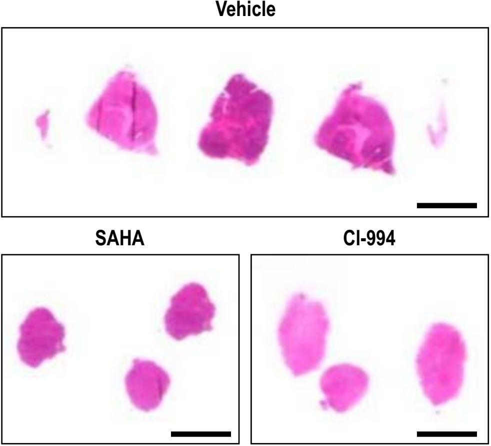
Vehicle
SAHA
CI-994

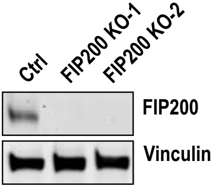
Ctrl
FIP200 KO-1
FIP200 KO-2
FIP200
Vinculin

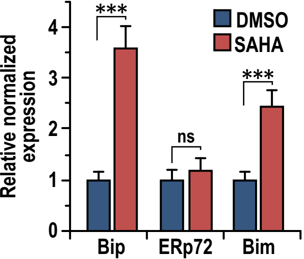

***
***
ns
DMSO
SAHA
Relative normalized expression
4
3
2
1
0
Bip
ERp72
Bim

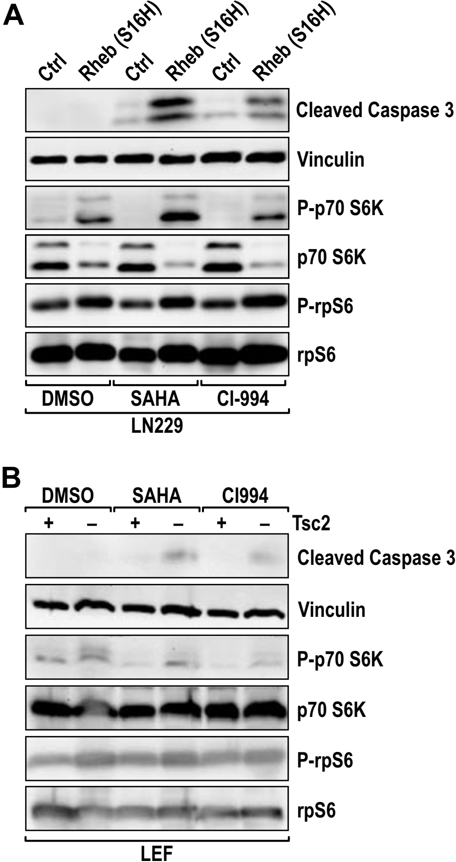
A
Ctrl
Rheb (S16H)
Ctrl
Rheb (S16H)
Ctrl
Rheb (S16H)
Cleaved Caspase 3
Vinculin
P-p70 S6K
p70 S6K
P-rpS6
rpS6
DMSO
SAHA
CI-994
LN229
B
DMSO
SAHA
CI994
+
–
+
–
+
–
Tsc2
Cleaved Caspase 3
Vinculin
P-p70 S6K
p70 S6K
P-rpS6
rpS6
LEF

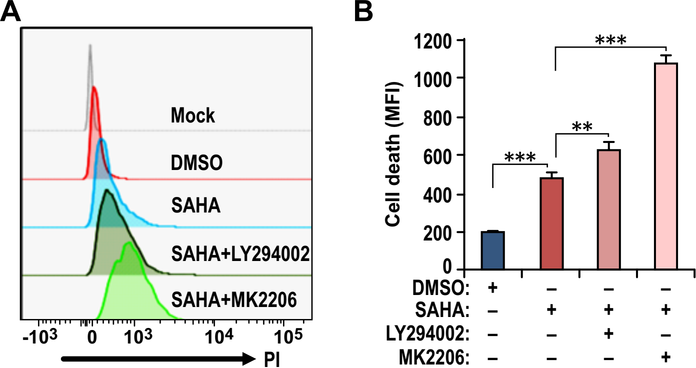
A
Mock
DMSO
SAHA
SAHA+LY294002
SAHA+MK2206
-10^3
0
10^3
10^4
10^5
PI
B
Cell death (MFI)
1200
1000
800
600
400
200
0
***
**
***
DMSO: + – – –
SAHA: – + + +
LY294002: – – + –
MK2206: – – – +
